# Efficacy and quality of life for FOLFOX/bevacizumab +/− irinotecan in first-line metastatic colorectal cancer—final results of the AIO CHARTA trial

**DOI:** 10.1038/s41416-023-02496-4

**Published:** 2023-11-23

**Authors:** Hans-Joachim Schmoll, Julia Mann, Fabian Meinert, Benjamin Garlipp, Kersten Borchert, Arndt Vogel, Eray Goekkurt, Ulrich Kaiser, Heinz-Gert Hoeffkes, Jörn Rüssel, Stephan Kanzler, Thomas Edelmann, Helmut Forstbauer, Thomas Göhler, Carla Hannig, Bert Hildebrandt, Carsten Roll, Carsten Bokemeyer, Jörg Steighardt, Franziska Cygon, Stefan Ibach, Alexander Stein, Joseph Tintelnot

**Affiliations:** 1 Clinic for Internal Medicine IV—Hematology/Oncology, University Clinic, Martin-Luther-University, Halle-Wittenberg, Germany; 2 Department of Medicine, Hematology and BMT with section Pneumology, University Cancer Center Hamburg, University Medical Center Hamburg-Eppendorf, Hamburg, Germany; 3 Department for Surgery, Oberhavel Kliniken Oranienburg, Oranienburg, Germany; 4 Clinic for Oncology/Hematology Klinikum Magdeburg, Magdeburg, Germany; 5 Clinic for Gastroenterology, Medical University Hannover, Hannover, Germany; 6 Clinic for Hematology/Oncology, St. Bernward Krankenhaus, Hildesheim, Germany; 7 Tumorclinic, Klinikum Fulda, Fulda, Germany; 8 Medical Clinic II, Leopoldina Clinic Schweinfurt, Schweinfurt, Germany; 9 Oncological Practice Schkeuditz, Schkeuditz, Germany; 10 Oncological Practice Rheinsieg, Bonn, Germany; 11 Oncological Center Dresden, Dresden, Germany; 12 Oncological Practice Bottrop, Bottrop, Germany; 13 Clinic for Gastroenterology, Hematology and Medical Oncology, Klinikum Barnim, Eberswalde, Germany; 14 Coordination Center for Clinical Trials Halle, Medical Faculty, Martin-Luther-University, Halle-Wittenberg, Germany; 15 X-act Cologne Clinical Research GmbH, Köln, Germany

**Keywords:** Colon cancer, Chemotherapy, Targeted therapies

## Abstract

**Background:**

FOLFOXIRI plus bevacizumab has demonstrated benefits for metastatic colorectal cancer (mCRC) patients. However, challenges arise in its clinical implementation due to expected side effects and a lack of stratification criteria.

**Methods:**

The AIO “CHARTA” trial randomised mCRC patients into clinical Group 1 (potentially resectable), 2 (unresectable/risk of rapid progression), or 3 (asymptomatic). They received FOLFOX/bevacizumab +/− irinotecan. The primary endpoint was the 9-month progression-free survival rate (PFSR@9). Secondary endpoints included efficacy in stratified groups, QoL, PFS, OS, ORR, secondary resection rate, and toxicity.

**Results:**

The addition of irinotecan to FOLFOX/bevacizumab increased PFSR@9 from 56 to 67%, meeting the primary endpoint. The objective response rate was 61% vs. 69% (*P* = 0.21) and median PFS was 10.3 vs. 12 months (HR 0.83; *P* = 0.17). The PFS was (11.4 vs. 12.9 months; HR 0.83; *P* = 0.46) in potentially resectable patients, with a secondary resection rate of 37% vs. 51%. Moreover, Group 3 (asymptomatic) patients had a PFS of 11.1 vs. 16.1 months (HR 0.6; *P* = 0.14). The addition of irinotecan did not diminish QoL.

**Conclusion:**

The CHARTA trial, along with other studies, confirms the efficacy and tolerability of FOLFOXIRI/bevacizumab as a first-line treatment for mCRC. Importantly, clinical stratification may lead to its implementation.

**Trial registration:**

The trial was registered as NCT01321957.

## Background

Despite the relevant improvements in the last decades, median survival in mCRC is limited to about 25–30 months in clinical trials. After negative trials combining a chemo-doublet with alternative angiogenetic inhibitors or the combination of bevacizumab and EGFR antibodies, the focus switched to triplet chemotherapy combinations with a targeted drug [1–15]. The CHARTA trial was initiated in parallel to other trials (TRIBE 1 + 2, STEAM, OLIVIA) to evaluate the addition of irinotecan to a FOLFOX and bevacizumab regimen in first-line mCRC [16–20]. Treatment guidelines for mCRC recommend the upfront stratification of patients based on the general constitution (e.g., co-morbidity or biological age) and disease-specific factors (e.g., number and extent of organ involvement or growth dynamics) to define the overall treatment aim [21]. The herein-used clinical grouping system categorises patients accordingly as Group 1: unresectable liver and/or lung metastases, potentially resectable after downsizing, comorbidities allowing surgery; Group 2: multiple metastases, rapid progression, risk of rapid deterioration, unlikely to become or never resectable; and Group 3: no symptoms or risk of rapid deterioration. However, its clinical application and the prognostic or predictive value have not been demonstrated yet. Consequently, the CHARTA trial was prospectively stratified to assess the clinical impact of such grouping in this disease setting. Furthermore, defining the right choice of treatment in this palliative setting requires to consider the quality of life (QoL) of the treated patients particularly with an intensified treatment regimen.

## Methods

### Study design and participants

The CHARTA trial (AIO KRK 0209) was an open-labelled, randomised, multicenter Phase II trial comparing FOLFOX/bevacizumab with or without irinotecan in patients with metastatic colorectal cancer. Patients were recruited from 51 centres in Germany.

Eligible patients were at least 18 years with a histologically confirmed unresectable metastatic colorectal cancer with or without primary tumour in situ. Patients were required to have measurable disease according to RECIST v1.1 (Response Evaluation Criteria in Solid Tumours) [22]. Other inclusion criteria were ECOG performance status (PS) of 0 to 2 (ECOG PS of 2, only if tumour-related), adequate baseline haematology and clinical chemistry. The study was conducted in accordance with the principles of good clinical practice and the Declaration of Helsinki. The study protocol was approved by the local ethics committees and was subject to authorisation by the competent authority. All participants provided written informed consent.

### Randomisation and masking

After obtaining informed consent, eligible patients were randomly assigned in a 1:1 ratio to FOLFOX and bevacizumab with or without irinotecan. Allocation was done centrally with a randomisation procedure and a stratification for the locally assessed clinical groups (1, 2 or 3) [21].

### Procedures

Screening assessments were completed within 28 days before the first dose of study treatment. The treatment was separated in two phases. The induction phase with a modified FOLFOX regimen (oxaliplatin at a dose of 85 mg/m^2^ iv over 2 h (day 1), LV at a dose of 200 mg/m^2^ iv over 2 h (day 1) and 5-FU at a dose of 3200 mg/m^2^ iv over 48 h (day 1–3)) and bevacizumab at a dose of 5 mg/kg iv over 30 to 90 min (day 1) with (FOLFOXIRI/bevacizumab) or without irinotecan (FOLFOX/bevacizumab) at a dose of 165 mg/m^2^ iv over 1 h (day 1) in a biweekly schedule for a maximum of 12 cycles (6 months) was followed by a maintenance phase with either 5-FU/LV and bevacizumab (same dosage and schedule as above) or capecitabine at a dose of 1600 mg/m^2^ in two doses orally day 1–14 and bevacizumab at a dose of 7.5 mg/kg iv over 30–90 min (day 1) every 3 weeks for up to 12 months. At the discretion of the investigator the first cycle of FOLFOXIRI/bevacizumab could be reduced to 75% of 5-FU/LV and irinotecan. Treatment was administered until progression, intolerable toxicity, secondary resection or for a maximum duration of 18 months. Dose modifications and reductions were based on toxicities causally related to the respective drug. Adverse events were coded according to the Common Terminology Criteria for Adverse Events (CTCAE) version 4.0. All patients who received at least one dose of the study drug were included in the safety analyses. Disease assessment was performed every 4 cycles (8 weeks) until 6 months (induction phase), followed by assessments every 3 month. Quality of life was assessed in parallel to disease assessment using the EORTC QLQ C30 and CR 29 questionnaire.

### Outcomes

The primary endpoint was the progression-free survival rate at 9 months (PFSR@9). Secondary endpoints included the efficacy in clinical groups, quality of life, progression-free survival (PFS), overall survival (OS), overall response rate (ORR) (according to RECIST v1.1), secondary resection rate and toxicity (according to NCI-CTCAE v4.0).

### Statistical analysis

The sample size calculation was based on a two-sided (continuity corrected) chi-square test for two independent groups, with PFSR@9 as the primary endpoint. First-line therapy with FOLFOX and bevacizumab displayed a median PFS of 9.4 months [23], leading to a PFSR@9 of ~55%. The four-drug combination treatment was expected to result in a PFSR@9 of at least 71.3%. The risk of estimating the four-drug combination treatment as active although the PFSR@9 was less than 71.3% should be 10%. The risk of rejecting the therapy although the PFSR@9 was more than 71.3% should be 20%, which leads to a power of 80%. With a dropout rate of 4% the number to be included was 125 patients per arm. Response rates and secondary resection rates were summarised using frequency tables and compared by logistic regression to adjust for the strata. For the time-to-event variables PFS and OS, the Kaplan–Meier method was used to estimate the event-free survival, and the log-rank test was conducted to compare the two treatment groups. Cox’s proportional hazard model was used to adjust for the influences of the three strata. Toxicity was documented in a descriptive manner. Quality of life was analysed according to the respective scoring manuals. For the mean comparison between treatment arms of respective clinical groups at the timepoint 0 or 24 weeks, the Mann–Whitney *U* Test was used. For the overall comparison of both treatment arms for clinically relevant changes (at least 10-point difference) of quality of life and dose reduction of chemotherapeutics, the two-sided chi-squared test was conducted. For comparisons of mean dose concentrations, the unpaired *t* test was used. The threshold for significance was set to *P* = 0.1.

## Results

### Patient characteristics

**Fig. 1:**
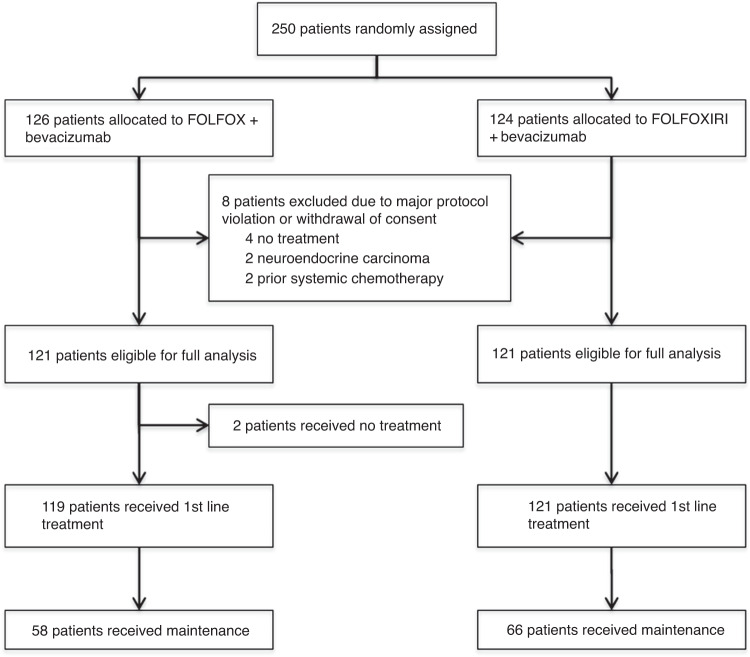
Consort diagram showing patients’ disposition. The number of assined, allocated, excluded, analyzed and treated patients are indicated.

Between July 2011 and December 2014, a total of 250 patients with mCRC were enrolled and randomised to FOLFOX/bevacizumab or FOLFOXIRI/bevacizumab. Overall, 6 patients received no treatment and thus, 244 patients were treated and formed the safety population. The modified (eligible) intent to treat (mITT) population comprised 242 patients after the exclusion of 8 patients due to withdrawal of consent before treatment (*n* = 4) and a major violation of selection criteria (neuroendocrine carcinoma *n* = 2 and prior systemic chemotherapy for metastatic disease *n* = 2). Patient disposition is displayed in the CONSORT diagram in Fig. 1. Baseline characteristics of the mITT population were well balanced between both treatment arms in the respective clinical groups (Table 1). The median age was 62 and 60 years, female gender was 35% and 36% of patients, and 95% and 97% of patients had ECOG 0/1, for the control compared to the experimental group, respectively. Notably, 90% and 83% of patients had synchronous metastases, and Köhne risk score was intermediate in 70% (in both arms) or high in 17% and 15%, indicating a prognostically rather poor patient population. Patients had overall RAS/BRAF wild-type tumours in 35% and left-sided primary tumours in 70%. The clinical group distribution was 29% in Group 1, 55% in Group 2 and 16% in Group 3. Clinical Group 3 patients had the highest median age of 64 or 68 years and the highest proportion of ECOG PS 2 patients with 11% and 5%, respectively.

**Table 1:** Patient characteristics (mITT) according to clinical groups.

Characteristic	Treatment arm	FOLFOX/bevacizumab, *N* = 121				FOLFOXIRI/bevacizumab, *N* = 121			
	Group (*n*)	ITT 1–3 (121)	1 (35)	2 (67)	3 (19)	ITT 1–3 (121)	1 (35)	2 (67)	3 (19)
Age: median (range)		60 (35–82)	59 (35–73)	60 (36–75)	68 (48–82)	62 (21–80)	61 (34–80)	61 (21–78)	64 (36–76)
Sex	Female/male	43 (36%)/78 (64%)	11/24 (31%/69%)	26/41 (39%/61%)	6/13 (32%/68%)	42 (35%)/79 (65%)	13/22 (37%/63%)	21/46 (31%/69%)	8/11 (42%/58%)
ECOG	0	62 (53%)	22 (63%)	30 (45%)	10 (53%)	56 (47.5%)	20 (57%)	26 (39%)	10 (53%)
	1	52 (44%)	12 (34%)	32 (48%)	8 (42%)	56 (47.5%)	13 (37%)	36 (54%)	7 (37%)
	2	3 (3%)		2 (3%)	1 (5%)	6 (5%)		4 (6%)	2 (11%)
	na	4 (3%)	1 (3%)	3 (4%)		4 (3%)	2 (6%)	1 (1%)	
Risk score (Köhne score)	Low	9 (7.5%)	6 (17%)	2 (3%)	1 (5%)	11 (9%)	5 (14%)	4 (65)	2 (11%)
	Intermediate	85 (70%)	25 (71%	47 70%)	13 (68%)	85 (70%)	25 (71%)	48 (72%)	12 (63%)
	High	18 (15%)	3 (9%)	12 (18%)	3 (16%)	20 (17%)	2 (6%)	13 (19%)	5 (26%)
	Missing	9 (7.5%)	1 (3%)	6 (9%)	2 (11%)	5 (4%)	3 (9%)	2 (3%)	
Mutational profile	BRAF V600E mutation	8 (7%)	3 (9%)	5 (7%)		5 (4%)		5 (7%)	
	Any RAS mutation	61 (50%)	14 (40%)	35 (52%)	12 (67%)	59 (49%)	16 (46%)	34 (51%)	9 (47%)
	BRAF/RAS wild-type	45 (37%)	16 (46%)	31 (46%)	6 (32%)	41 (34%)	14 (40%)	24 (36%)	9 (47%)
	Missing	7 (6%)	5 (14%)	1 (1%)	1 (5%)	16 (13%)	5 (14%)	9 (13%)	1 (5%)
Timepoint of metastases	Synchronous	110 (90%)	28 (80%)	66 (99%)	16 (84%)	101 (83%)	28 (80%)	59 (88%)	14 (74%)
	Metachronous	11 (10%)	7 (20%)	1 (1%)	3 (16%)	20 (17%)	7 (20%)	8 (12%)	5 (26%)
Primary tumour location	Left	84 (69%)	27 (77%)	46 (69%)	11 (56%)	88 (73%)	28 (80%)	45 (67%)	15 (79%)
	Right	36 (30%)	8 (23%)	20 (31%)	8 (44%)	28 (23%)	5 (14%)	19 (28%)	4 (21%)
	Missing	1 (1%)		1 (1%)		5 (4%)	2 (6%)	3 (5%)	

*ECOG* Eastern Cooperative Oncology Group performance status, *na* not available, *n* number.

### Treatment intensity and duration

**Fig. 2:**
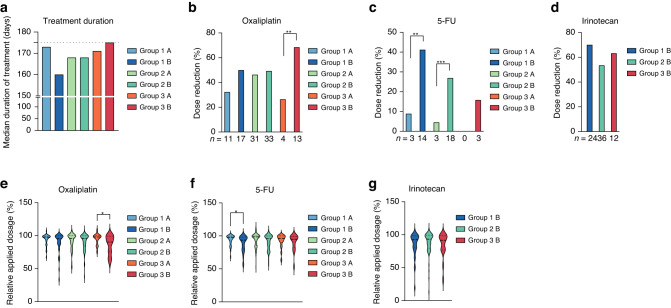
Dose intensity and duration of treatment according to clinical groups. **a** Time of treatment is shown as the median time of treatment in days per treatment arm. Dose reductions (-at least 5% of medication) during the induction phase of the study are shown as relative per treatment arm for oxaliplatin (**b**), 5-FU (**c**) or irinotecan (**d**). The relative applied dosage of oxaliplatin (**e**), 5-FU (**f**) or irinotecan (**g**) was calculated as relative to the maximal applied dosage per protocol and patient. *P < 0.05, **P < 0.001, ***P < 0.0001, determined by two-tailed chi-square test (**b**, **c**) or unpaired *t* test (**e**–**g**). Arm A is FOLFOX/bevacizumab and arm B FOLFOXIRI/bevacizumab.

The median duration of the induction phase treatment was 5.6 months (range 0.5–13) in both arms, ranging between median 5.3 months in clinical Group 1 (potentially resectable) and 5.8 months in clinical Group 3 (asymptomatic) patients (Fig. 2a). The median number of cycles administered was similar in both arms with 12 cycles (range 1–12) in the induction phase (first 6 months of treatment) and 8 cycles (range 1–26) in the maintenance phase. Dose reductions were significantly more often required for FOLFOXIRI/bevacizumab (8.9% vs. 17.1% of cycles, *P* < 0.00001) considering the total mITT population.

Particularly, clinical Group 1 and 2 patients receiving FOLFOXIRI/bevacizumab required more 5-FU dose reductions during the induction phase (*P* = 0.0021 and *P* = 0.0004; Fig. 2b) and clinical Group 3 patients required more oxaliplatin dose reductions (*P* = 0.0093; Fig. 2c). Irinotecan was reduced in 70% (Group 1), 53% (Group 2) or 63% (Group 3) of patients (Fig. 2d). The per protocol defined initial dose reduction was applied in 21 patients (Group 1: 10 patients (14%), Group 2: 10 patients (7.4%) and Group 3: 1 patient (2.6%)) and maintained in 13 patients in the second and third cycle. In contrast to Groups 1 and 2, the mean applied dosage of oxaliplatin (95%) was higher in Group 3 patients treated with FOLFOX/bevacizumab compared to FOLFOXIRI/bevacizumab (86%, *P* = 0.0198; Fig. 2e). In Group 1 patients, the mean applied dosage of 5-FU was higher with FOLFOX/bevacizumab (94%) compared to FOLFOXIRI/bevacizumab (89%, *P* = 0.0318; Fig. 2f) and the mean irinotecan dosages where comparable in all the groups treated with irinotecan (Fig. 2g).

### Efficacy

**Fig. 3:**
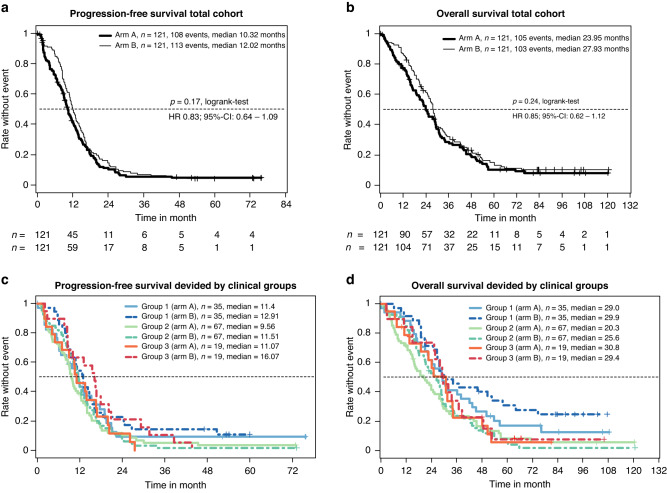
PFS and OS in total study cohort or divided by clinical Groups. **a** The Kaplan–Meier estimator for the respective treatment arm of the total mITT population is shown. The number of patients per arm and timepoint and median PFS interval is indicated. **b** As in (**a**), except OS is shown for respective arms. **c** The Kaplan–Meier estimator for respective clinical groups and treatment arms is shown. The number of patients per group and median PFS interval is indicated. **d** As in (**a**), except OS is shown for respective groups. Arm A is FOLFOX/bevacizumab and arm B FOLFOXIRI/bevacizumab.

After a median follow-up of 87.6 months (inverse Kaplan–Meier method), the efficacy was determined in the mITT population of eligible patients (*n* = 242). The progression-free survival rate at 9 months was significantly improved from 56.2% with FOLFOX/bevacizumab to 66.9% with FOLFOXIRI/bevacizumab on the predetermined significance level of 10% (*P* = 0.086—stratified logistic regression; Fig. 3a and Table 2). Thus, the primary endpoint was met. The objective response rate (ORR) was 61% and 69% (*P* = 0.21), secondary R0/1-resection rate was 15% vs. 20% (*P* = 0.39) and with the inclusion of patients with complete remission (CR) the rate was 18% vs. 24% (*P* = 0.34). Median PFS was 10.3 vs. 12.0 months (HR 0.83, 95% CI 0.64–1.08, *P* = 0.19), and the median OS was 24.0 vs. 28.0 months (HR 0.82, 95% CI 0.62–1.09, *P* = 0.24), for control and experimental group, respectively (Table 2 and Fig. 3b). Subsequent second-line treatments were applied in 67.8% vs. 73.6% of patients, and third line treatments in 40.5% vs. 43.8% of patients (Supplementary Table A1). The type of salvage regimen was comparable in both groups.

**Table 2:** Efficacy according to RECIST 1.1.

Efficacy parameters		FOLFOX/bevacizumab (*N* = 121)	FOLFOXIRI/bevacizumab (*N* = 121)	HR (95% CI)	*P* value
PFS rate at 9 months		56.2%	66.9%	–	**0.086**^§^
PFS		10.3 months	12.0 months	0.84 (0.64–1.09)	0.19*
OS		24.0 months	28.0 months	0.85 (0.62–1.12)	0.24*
CR + PR		69 (60.5%)	79 (69.3%)	–	0.21^#^
CR		6 (5.3%)	6 (5.3%)		
PR		63 (55.3%)	73 (64%)		
Sec. R0/1 resection		15%	20%	–	0.39^#^
Group 1 (potentially resectable)	PFS	11.4 months	12.9 months	0.83	0.46
	OS	29.0 months	29.9 months	0.73	0.23
	RR	56.2%	67.6%	–	0.45
	Sec. R0/1 resection	37.1%	51.4%	–	0.33
	GHS mean ± SD (n) at w24	62.9 ± 12.6 (11)	53.6 ± 16.6 (7)	–	0.156^$^
Group 2 (unresectable/risk of rapid progression)	PFS	9.6 months	11.5 months	0.94	0.73
	OS	20.3 months	25.6 months	0.95	0.78
	RR	62.5%	67.7%	–	0.58
	Sec. R0/1 resection	6.0%	7.5%	–	1.0
	GHS mean ± SD (n) at w24	65.2 ± 15.8 (22)	61.6 ± 19.8 (28)	–	0.556^$^
Group 3 (asymptomatic, slow progress)	PFS	11.1 months	16.1 months	0.6	0.14
	OS	30.8 months	29.4 months	0.84	0.62
	RR	61.1%	77.8%	–	0.47
	Sec. R0/1 resection	6%	7.5%	–	1.0
	GHS mean ± SD (*n*) at w24	50 ± 13.9 (6)	64.2 ± 22.6 (10)	–	0.185^$^

*n* number, *PFS* progression-free survival, *OS* overall survival, *ORR* overall response rate, *CR* complete response, *PR* partial response, *CI* confidence interval, *SD* standard deviation.

Test methods (two-sided): ^§^stratified logistic regression, *logrank-test, ^#^Fisher’s exact test, ^$^Mann–Whitney *U* Test).

Significant *P* values are highlighted.

### Subgroup analyses

The clinical grouping, which was determined by the investigators and used as the stratification factor for randomisation, worked well to discriminate the three groups with different prognoses (Table 2 and Fig. 3c, d) and to differentiate the comparative effect of both treatment arms. Although not significantly different, the PFS benefit of the addition of irinotecan was mainly driven by clinical Group 3 (HR 0.6; *P* = 0.14) and to a bit lesser extent by clinical Group 1 (HR 0.83; *P* = 0.46), but very little by clinical Group 2 (HR 0.94; *P* = 0.73) (Table 2). In line with the improved PFSR@9, FOLFOXIRI/bevacizumab led to a trend of increased secondary R0/1-resection rates in clinical Group 1 patients from 37.1% to 51.4% (*P* = 0.33). Expectedly, patients achieving a “No Evidence of Disease” (NED) status by secondary resection or chemotherapy alone had a nearly doubled median OS (44.2 vs. 23.8 months, *P* < 0.0001) and showed a trend to improved PFS (*P* = 0.013) compared to patients not achieving NED status (Supplementary Fig. A1). Considering the mITT age below 61 (HR 0.57) and synchronous metastasis (HR 0.75) favoured irinotecan. Furthermore, a trend towards improved PFS in the FOLFOXIRI/bevacizumab arm was observed for RAS wild-type (HR 0.74) and left colon (HR 0.76), but not ECOG or BRAF status (Supplementary Fig. A2).

### Toxicity

Treatment was generally given outpatient and was well tolerated. Adverse events (AE) are summarised in Supplementary Table A2. The grade 3/4 adverse events, which were numerically higher in the experimental arm were: neutropenia (14% vs. 20%), with only 1% febrile neutropenia in both arms, diarrhoea (12% vs. 16%), fatigue/asthenia (3% vs. 9%), treatment-related fatal (*n* = 3 vs. 1) and overall fatal (*n* = 5 vs. 2). Overall, treatment with FOLFOXIRI/bevacizumab was feasible, and the most occurring adverse events were mild to moderate. The adverse event-related discontinuation rate was 16.5% vs. 19.8%, the total serious adverse event rate was 85% vs. 89% and the total fatal outcome was 3 vs. 4, comparing FOLFOX/bevacizumab and FOLFOXIRI/bevacizumab.

### Quality of life

**Fig. 4:**
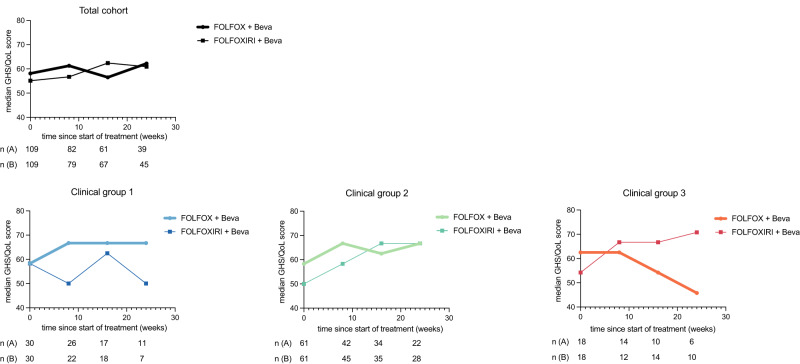
Median GHS/QoL in total population or clinical Groups 1, 2 and 3 during treatment with FOLFOX/bevacizumab +/− irinotecan. Median GHS scores were calculated as described in “Methods”. Number of patients per group and treatment arm are indicated. Median GHS scores were reported for total mITT population or respective clinical groups and treatment arms at timepoints 0, 8, 16 or 24 weeks of treatment.

Health-related quality of life questionnaires were available from 95.4% at baseline, 72.6% at week 8, 59.5% at week 16 and 43.5% at week 24. No significant difference in the median Global health score (GHS) at week 0, 8, 16 and 24 were seen between both total population treatment groups (Fig. 4). Moreover, the GHS mean values pooled over all induction timepoints were similar between treatment groups (59.8 and 58.8, *P* = 0.726) (Supplementary Table A3). Clinically relevant changes in GHS score (at least 10 points) were similar between both groups, showing an improvement in 44.3% and 39.7% (*P* = 0.629) and deterioration in 21.5% and 26.9% (*P* = 0.461) for the control compared to the experimental group, respectively (Supplementary Table S4). No significant difference was observed in either clinical group comparing both treatment arms, however, the GHS mean values showed a trend towards improvement at a clinically relevant difference during therapy for patients treated with FOLFOXIRI/bevacizumab among clinical Group 3 patients (> 10 points, *P* = 0.185; Fig. 4).

## Discussion

The randomised CHARTA trial tested the addition of irinotecan to FOLFOX/bevacizumab in first-line metastatic colorectal cancer treatment and met its primary endpoint of an improved PFS rate at 9 months. In addition, numerically albeit not statistically significant improvements in ORR, PFS and OS were noted in favour of FOLFOXIRI/bevacizumab, in line with the results of other trials in this setting [24–27]. The control arm with the chemotherapy doublet and bevacizumab seemed to perform slightly better in “CHARTA” with a median PFS of 10.3 months compared to 9.7 months for FOLFIRI/ bevacizumab in “TRIBE” and 9.3 for FOLFOX/ bevacizumab in “STEAM” based on which the PFSR@9 month was selected as the primary endpoint. Thus, the hazard ratio for PFS with 0.84 is less pronounced in the “CHARTA” trial compared to “TRIBE 1 and 2” (HR 0.75). In addition, disease assessments were scheduled differently in “CHARTA”, every 2 months for 6 months, followed by 3 monthly assessments, compared to “TRIBE2”, where the assessments were conducted every 8 weeks even during the maintenance regimen. Furthermore, patient characteristics differed between the “TRIBE” trials and “CHARTA” particular regarding ECOG PS distribution (ECOG 0 90% vs. 50%). Overall, these small differences might have contributed to the slight difference in the hazard ratios and may explain why only the primary endpoint of PFSR@9 was different but not ORR, PFS and OS.

Even though the effectiveness of FOLFOXIRI/bevacizumab in treating mCRC is well-established, only a small percentage of all mCRC patients (less than 5%) and mCRC patients over the age of 50 (less than 2%) are currently receiving this combination therapy [28]. The CHARTA trial’s patient stratification based on predicted clinical outcomes presents an opportunity to customise treatments and ensure the implementation of this regimen in clinical practice. We prospectively categorised patients into clinical groups based on treatment aims (Group 1: potentially resectable after downsizing; Group 2: unlikely to become or never resectable; and Group 3: no symptoms or risk of rapid deterioration). Although we did not observe a significant difference in PFS or OS in either group, we observed a trend for the greatest PFS benefit in clinical Group 3 patients (HR 0.6). However, guidelines recommend a more conservative, sequential approach for this group due to factors such as absence of symptoms, advanced age, frailty, or lack of eligibility for post-chemotherapy metastasis resection. This benefit might be linked to a low rate of 5-FU dose reductions in this group (Fig. 2a, c, f). Considering OS, clinical Group 1 patients within the FOLFOXIRI/bevacizumab arm contained an extraordinary overall survival rate after 5 years. This is closely related to the achievement of a NED status (Fig. 3d and Table 1). Notably, clinical Group 2 patients derived no apparent benefit of the addition of irinotecan to FOLFOX/bevacizumab in terms of efficacy, however, also did not experience any decline in their QoL. In CHARTA, the clinical Group 2 (unresectable, symptomatic or risk of rapid progression) was larger than expected (55.3%) likely due to the selection of advanced patients for clinical trials testing the intensified regimen.

Besides clinical groups, other tumour-specific or patient’s specific factors correlated with improved progression-free survival such as “RAS wt status”, primary tumour sidedness, synchronous metastasis or age which is in line with other studies testing FOLFOXIRI/bevacizumab [26].

The main limitation of these results are the overall low numbers of patients who are particularly considered in the subgroup analysis and specifically as clinical Group 3. Hence, it is important to acknowledge the possibility that chance may have influenced the favourable outcomes observed in clinical Group 3. Consequently, additional studies are warranted to investigate the potential suitability of intensified treatment for patients with older age and asymptomatic disease.

The FOLFOXIRI/bevacizumab regimen demonstrated good tolerability with no unexpected safety findings. Dose modifications were primarily necessitated by haematological (36.4%) or gastrointestinal (26.7%) toxicity. The primary grade 3/4 adverse events experienced by patients on this combination included diarrhoea, fatigue, and nausea. However, these differences did not translate into noticeable impacts on patient-reported global health scores, suggesting only a minimal effect on overall QoL.

In summary, we provide further data showing that FOLFOXIRI/bevacizumab is superior to FOLFOX/Bevacizumab considering PFSR@9 in first-line mCRC treatment. Moreover, patient stratification according to clinical grouping is feasible and potentially defines patients who are more likely to benefit from the treatment. Such a classification may help to implement this effective but rather aggressive treatment regimen into clinical practice.

## Conclusion

This multicentre randomised trial demonstrates a superior PFS rate at 9 months for the addition of irinotecan to FOLFOX/bevacizumab, with a good tolerability and QoL profile, and thus supports the value of FOLFOXIRI/bevacizumab in first-line treatment of mCRC. Furthermore, findings from the CHARTA trial suggest a clinical stratification that could identify patients who might particularly benefit from this regimen.

### Supplementary information

Supplementary tables and figures

CONSORT Checkliste

## Data Availability

The datasets used and/or analysed during the current study are available from the corresponding author on reasonable request.
