# Supplementary material for: Efficacy and quality of life for FOLFOX/bevacizumab +/− irinotecan in first-line metastatic colorectal cancer—final results of the AIO CHARTA trial
Source: Br J Cancer. 2023 Nov 23;130(2):233–41. doi: 10.1038/s41416-023-02496-4 (PMC10803799; doi:10.1038/s41416-023-02496-4)
Supplement: Supplementary file 1 — Supplementary tables and figures [file 41416_2023_2496_MOESM1_ESM.docx]

**Appendices (tables and figures)**

**Table A1: Further line treatments**

|  | FOLFOX/bevacizumab | | FOLFOXIRI/bevacizumab | |
| --- | --- | --- | --- | --- |
|  | **N** | **%** | **N** | **%** |
| **Any second-line therapy** | **82** | **67.8** | **89** | **73.6** |
| **Chemotherapy with anti-EGFR** | 21 | 25.6 | 19 | 21.3 |
| **RAS wildtype patients**  **treated with anti-EGFR/overall** | 16/32 | 50 | 17/38 | 44.7 |
| **Chemotherapy with anti-VEGF** | 34 | 41.5 | 44 | 49.4 |
| **Chemotherapy doublet regimen without anti-VEGF/EGFR** | 11 | 13.4 | 10 | 11.2 |
| **Chemotherapy single agent** | 11 | 13.4 | 4 | 4.5 |
| **anti-VEGF/EGFR single agent** | 4 | 4.9 | 9 | 10.1 |
| **Other** | 1 | 1.2 | 3 | 3.4 |
| **Any third-line therapy** | **49** | **40.5** | **53** | **43.8** |
| **Chemotherapy with anti-EGFR** | 6 | 12.2 | 12 | 22.6 |
| **RAS wildtype patients**  **treated with anti-EGFR/overall** | 6/18 | 33.3 | 12/23 | 52.2 |
| **Chemotherapy with anti-VEGF** | 20 | 40.8 | 20 | 37.7 |
| **Chemotherapy doublet regimen without anti-VEGF/EGFR** | 6 | 12.2 | 3 | 5.7 |
| **Chemotherapy single agent** | 4 | 8.2 | 5 | 9.4 |
| **anti-VEGF/EGFR signle agent** | 12 | 13.3 | 12 | 22.6 |
| **Other** | 1 | 2.0 | 1 | 1.9 |

**Table A2: Grade 3/4 adverse events and SAEs**

|  | FOLFOX/bevacizumab **(N=121)** | FOLFOXIRI/bevacizumab **(N=121)** |
| --- | --- | --- |
|  | | |
| **Toxicity** | | |
|  | | |
| **Grade 3/4 adverse events** | | |
| **Diarrhea** | 12% | 16% |
| **Nausea** | 3% | 8% |
| **Vomiting** | 3% | 3% |
| **Mucositis** | 3% | 3% |
| **Neutropenia** | 14% | 20% |
| **Febrile Neutropenia** | 1% | 1% |
| **Infection** | 12% | 10% |
| **Hypertension** | 7% | 9% |
| **Neuropathy** | 4% | 3% |
| **Pulmonary embolism** | 3% | 2% |
| **Fatigue/Asthenia** | 3% | 9% |
|  | | |
| **SAEs** | | |
| **Overall (n pts)** | 85 (51) | 89 (49) |
| **Fatal overall** | 2 | 5 |
| **Fatal treatment related** | 1 | 3 |

**Table A3: Comparison of EORTC QLQ C30 and CR29 scales pooled over induction period**

| **Scales** | FOLFOX/bevacizumab **mean value ± SD** | FOLFOXIRI/bevacizumab  **mean value ± SD** | **p-value (CI 95%)** |
| --- | --- | --- | --- |
| **EORTC QLQ C30** |  |  |  |
| Global health status | 59.8 ± 18.8 | 58.8 ± 18.9 | 0.726 (-4.60 – 6.60) |
| Physical functioning | 76.7 ± 21.6 | 76.2 ± 24.2 | 0.889 (-6.45 – 7.42) |
| Role functioning | 64.9 ± 29.1 | 63.0 ± 32.2 | 0.686 (-7.31 – 11.10) |
| Emotional functioning | 73.3 ± 23.4 | 70.3 ± 20.8 | 0.335 (-3.36 – 9.31) |
| Cognitive functioning | 86.1 ± 18.0 | 83.5 ± 20.1 | 0.368 (-3.10 – 8.33) |
| Social functioning  functioning | 69.6 ± 26.9 | 66.0 ± 28.3 | 0.390 (-4.67 – 11.91) |
| Fatigue | 40.4 ± 23.6 | 41.1 ± 24.5 | 0.842 (-7.94 – 6.48) |
| **Nausea and vomiting** | **9.4 ± 13.9** | **16.0 ± 20.7** | **0.015 (-11.84 – (-1.31)** |
| Pain | 21.7 ± 25.9 | 23.9 ± 25.6 | 0.569 (-9.98 – 5.50) |
| Dyspnea | 22.3 ± 26.2 | 25.2 ± 27.7 | 0.484 (-10.98 – 5.22) |
| Insomnia | 27.8 ± 27.7 | 32.0 ± 27.6 | 0.321 (-12.48 – 4.11) |
| Appetite loss | 22.3 ± 25.3 | 27.7 ± 28.3 | 0.186 (-13.50 – 2.63) |
| Constipation | 11.0 ± 19.5 | 9.9 ± 16.8 | 0.689 (-4.36 – 6.59) |
| **Diarrhea** | **23.7 ± 26.6** | **32.1 ± 28.5** | **0.051 (-16.50 – 0.04)** |
| Financial difficulties | 21.0 ± 28.3 | 28.3 ± 29.6 | 0.095 (-16.08 – 1.28) |
| **EORTC QLQ CR29** |  |  |  |
| Body image | 78.5 ± 24.4 | 78.4 ± 22.6 | 0.971 (-6.99 – 7.26) |
| Anxiety | 52.2 ± 27.4 | 51.3 ± 28.5 | 0.834 (-7.56 – 9.36) |
| Weight | 76.2 ± 28.5 | 68.5 ± 26.8 | 0.069 (-0.62 – 16.16) |
| Sexual function: men | 34.3 ± 25.9 | 36.2 ± 23.2 | 0.697 (-11.03 – 7.39) |
| Sexual function: women | 16.9 ± 18.6 | 12.6 ± 21.7 | 0.439 (-6.65 – 15.11) |
| Urinary frequency | 36.1 ± 27.0 | 35.2 ± 23.0 | 0.809 (-6.67 – 8.54) |
| Blood and mucus in stool | 5.7 ± 14.4 | 7.0 ± 11.9 | 0.539 (-5.24 – 2.75) |
| Urinary incontinence | 4.0 ± 11.7 | 4.4 ± 10.6 | 0.790 (-3.85 – 2.94) |
| Dysuria | 3.3 ± 12.4 | 5.6 ± 14.0 | 0.259 (-6.31 – 1.71) |
| Abdominal pain | 17.8 ± 23.7 | 15.8 ± 20.5 | 0.554 (-4.71 – 8.75) |
| Buttock pain | 13.1 ± 22.2 | 18.8 ± 22.2 | 0.095 (-12.44 – 1.00) |
| Bloated feeling | 16.1 ± 21.3 | 17.8 ± 22.3 | 0.608 (-8.36 – 4.90) |
| Dry mouth | 32.2 ± 29.2 | 29.7 ± 25.6 | 0.556 (-5.87 – 10.87) |
| **Hair loss** | **27.7 ± 27.5** | **36.8 ± 28.9** | **0.029 (-18.09 – (-1.01))** |
| Trouble with taste | 39.1 ± 32.4 | 38.9 ± 30.1 | 0.958 (-922 – 9.73) |
| Impotence | 40.1 ± 30.9 | 41.9 ± 31.2 | 0.768 (-13.75 – 10.19) |
| Dyspareunia | 5.8 ± 11.3 | 4.5 ± 15.6 | 0.765 (-7.43 – 10.04) |
| Stool frequency with stoma | 25.1 ± 19.4 | 16.9 ± 17.3 | 0.132 (-2.56 – 19.01) |
| **Stool frequency without stoma** | **19.1 ± 18.9** | **28.1 ± 25.6** | **0.030 (-16.98 – (-0.87))** |
| Bloating with stoma | 28.7 ± 20.0 | 21.1 ± 17.6 | 0.169 (-3.39 – 18.75) |
| Bloatung without stoma | 22.8 ± 26.6 | 22.8 ± 25.1 | 0.989 (-9.25 – 9.37) |
| Faecal incontinence with stoma | 31.0 ± 29.0 | 10.9 ± 17.3 | 0.007 (5.71 – 34.46) |
| Faecal incontinence without stoma | 5.5 ± 19.0 | 10.2 ± 22.7 | 0.218 (-12.22 – 2.82) |
| Sore skin with stoma | 34.1 ± 29.9 | 58.8 ± 18.9 | 0.110 (-2.96 – 28.02) |
| **Sore skin without stoma** | **14.2 ± 23.6** | 76.2 ± 24.2 | **0.034 (-19.26 – (-0.77))** |
| Embarrassed by bowel movement with stoma | 41.5 ± 34.7 | 63.0 ± 32.2 | 0.086 (-2.44 – 35.31) |
| Embarrased by bowel movement without stoma | 8.6 ± 21.5 | 70.3 ± 20.8 | 0.408 (CI -11.63 – 4.76) |
| Stoma care problems | 23.9 ± 30.9 | 83.5 ± 20.1 | 0.033 (CI 1.34 – 29.36) |

(Abbrev: SD standard deviation, CI confidence interval)

**Table A4: Clinically relevant changes in EORTC QLQ C30 scales between baseline and week 8**

|  | **FOLFOXBev**  **n (%)** | **FOLFOXIRIBev**  **n (%)** | **p-value** | **FOLFOXBev**  **n (%)** | **FOLFOXIRIBev n (%)** | **p-value** |
| --- | --- | --- | --- | --- | --- | --- |
| **Patients** | 79****** | 78****** |  | 79****** | 78****** |  |
|  | **Increase of at least 10 points***  **(improvement)** | | | **Decrease of at least 10 points***  **(deterioration)** | | |
| **Global health status** | 35 (44.3%) | 31 (39.7%) | 0.629 | 17 (21.5%) | 21 (26.9%) | 0.461 |
| **Physical functioning** | 27 (34.2%) | 34 (43.6%) | 0.254 | 20 (25.3%) | 19 (24.4%) | 1.00 |
| **Role functioning** | 26 (33.3%) | 27 (35.1%) | 0.866 | 29 (37.2%) | 24 (31.2%) | 0.499 |
| **Emotional functioning** | 40 (50.6%) | 35 (44.9%) | 0.524 | 15 (19%) | 18 (23.1%) | 0.562 |
| **Cognitive functioning** | 12 (15.2%) | 13 (16.7%) | 0.830 | 20 (25.3%) | 21 (26.9%) | 0.857 |
| **Social functioning** | 27 (34.2%) | 17 (21.8%) | 0.110 | 22 (27.8%) | 25 (32.1%) | 0.604 |
|  | **Increase of at least 10 points***  **(deterioration)** | | | **Increase of at least 10 points***  **(deterioration)** | | |
| **Fatigue** | 34 (43.2%) | 30 (39.0%) | 0.629 | 25 (31.6%) | 31 (40.3%) | 0.317 |
| **Nausea and vomiting** | 25 (31.6%) | 34 (44.2%) | 0.137 | 10 (12.7%) | 12 (15.6%) | 0.651 |
| **Pain** | 18 (22.8%) | 16 (20.5%) | 0.847 | 28 (35.4%) | 29 (37.2%) | 0.869 |
| **Dyspnea** | 17 (21.8%) | 17 (22.7%) | 1.00 | 11 (14.1%) | 20 (26.7%) | 0.070 |
| **Insomnia** | 17 (21.5%) | 20 (26.0%) | 0.574 | 20 (25.3%) | 20 (26.0%) | 1.00 |
| **Appetite loss** | 15 (19.2%) | 27 (35.1%) | 0.031 | 16 (20.5%) | 18 (23.4%) | 0.701 |
| **Constipation** | 10 (12.7%) | 10 (12.8%) | 1.00 | 7 (8.9%) | 11 (14.1%) | 0.328 |
| **Diarrhea** | 28 (35.9%) | 33 (42.3%) | 0.512 | 19 (24.4%) | 12 (15.4%) | 0.228 |
| **Financial difficulties** | 13 (16.7%) | 23 (30.3%) | 0.057 | 11 (14.1%) | 7 (9.2%) | 0.453 |

*Increase or decrease of at least 10 points between baseline and week 8

** Based on the completeness of data from the questionnaires patient numbers range between 75 and 79, displayed by the rate (%)

**figure A1**


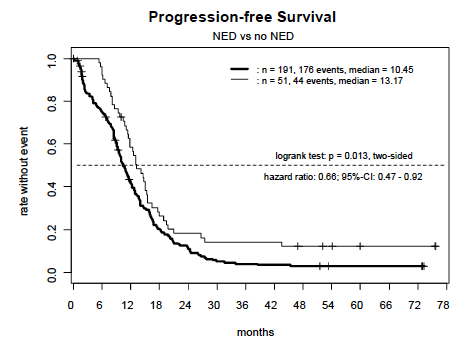

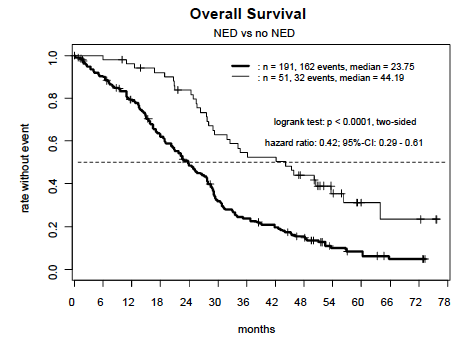


A

B

**figure A1: Subgroup analysis for PFS and OS for achievement of No Evidence of Disease status (NED) defined as secondary R0/1 resection or complete remission on imaging vs. no NED status.** (A) The Kaplan-Meier estimator for respective NED status of the total mITT population is shown. Number of patients per arm and median PFS interval is indicated. (B) as in A, except OS is shown for respective arms. Thick line indicates no NED and thin line NED.

**figure A2**

**
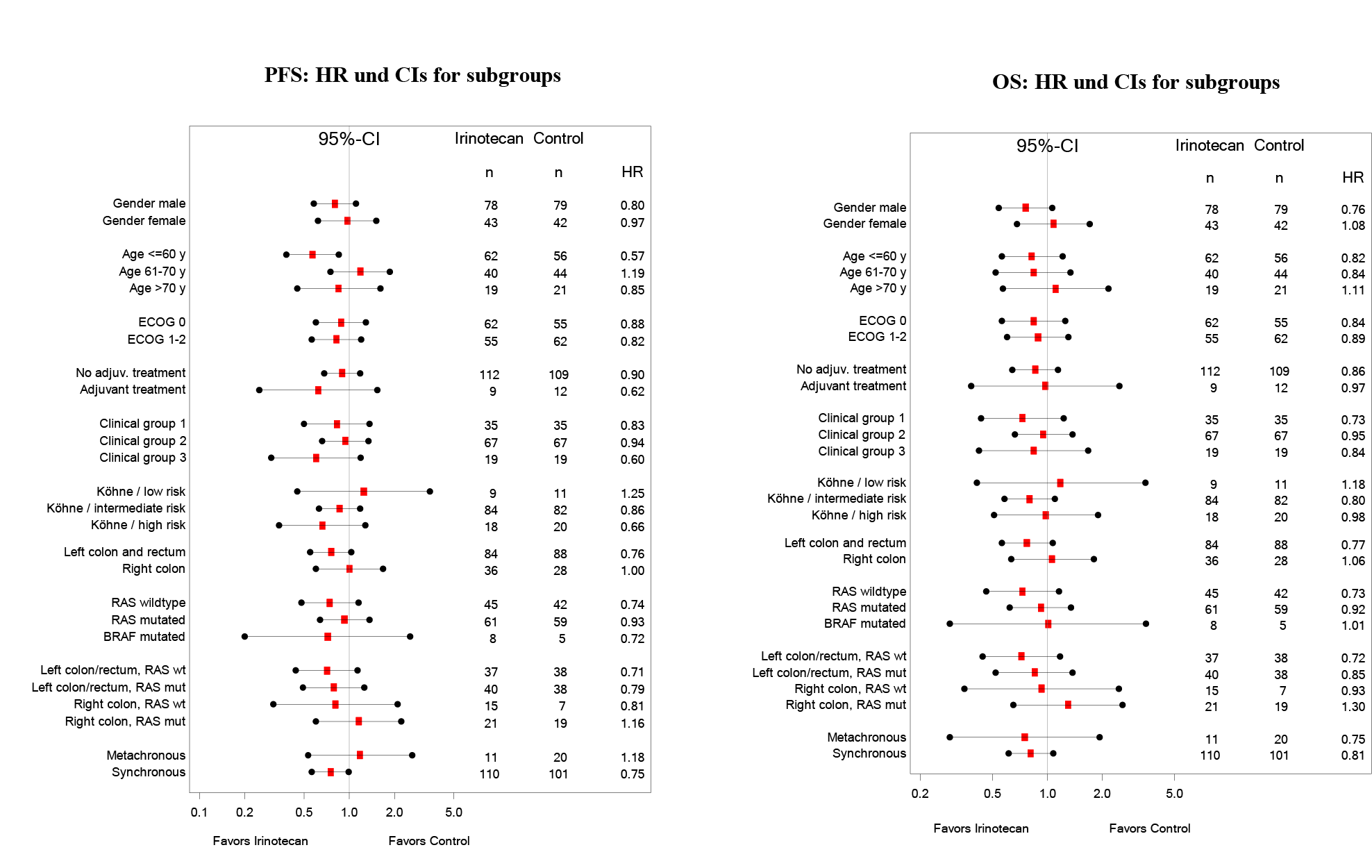
**

**figure A2: Forest plot effect of clinico-pathological factors on PFS (left) and OS (right).**
